# Assessment of Knowledge, Attitude, and Practice of Skilled Assistance Seeking Maternal Healthcare Services and Associated Factors among Women in West Shoa Zone, Oromia Region, Ethiopia

**DOI:** 10.1155/2021/8888087

**Published:** 2021-08-10

**Authors:** Eden Girmaye, Kassa Mamo, Birhanu Ejara, Fikadu Wondimu, Maru Mossisa

**Affiliations:** Department of Midwifery, College of Medicine and Health Sciences, Ambo University, Ambo, Ethiopia

## Abstract

**Background:**

This study aimed to assess women's knowledge, attitude, and practice towards skilled assistance seeking maternal healthcare services in West Shoa Zone, Oromia Region, Ethiopia.

**Methods:**

Cross-sectional survey design was conducted from 1 February to 23 March 2018 in West Shoa Zone, Oromia, Ethiopia. A simple random sampling technique was used to select the participants. The data were collected using a pretested and structured questionnaire. Data were entered using EpiData version 3.1, and descriptive analysis and bivariate and multivariate logistic regression analyses were carried out using SPSS version 20 statistical software package.

**Results:**

The study revealed that the knowledge, attitude, and practice towards skilled maternal health services were found such that 473.3 (72.4%) of the study participants had good knowledge, 180.7 (27.6%) had poor knowledge, and 400 (61.3%) had positive attitude, 254 (38.84%) had negative attitude, 460.3 (70.4%) had good practice, and 193.7 (29.6%) had poor practice towards skilled maternal health services. Factors that had a significant association with antenatal care utilization were planned pregnancy (AOR = 8.2, 95% CI = 3.39-19.78-0.87) and access to transport (AOR = 3.1, 95% CI = 1.46–6.61). Attending ANC at least once (AOR = 3.1, 95% CI = 1.13–8.41), women's education (AOR = 3.0, 95% CI = 1.18–7.84), and unplanned pregnancy (AOR = 0.3, 95% CI = 0.21–0.75) were factors associated with skilled delivery service utilization. Attending ANC at least once (AOR = 2.1, 95% CI = 1.1–4.2), birth complications (AOR = 2.2, 95% CI = 1.35–3.66), unplanned pregnancies (AOR = 0.3, 95% CI = 0.22–0.68), and awareness about skilled obstetric care (AOR = 3.7, 95% CI = 1.68–12.79) were factors associated with postnatal care utilization.

**Conclusions:**

This study found that the knowledge, attitude, and practice of skilled maternal health services among the study participants are low, showing less than three-quarters of the total sample size. Therefore, this study implied that interventions are required to improve women's knowledge, attitude, and practice of skilled maternal health services in the study area. Furthermore, women's education is significantly associated with skilled delivery service utilization. Accordingly, this study recommends that improving equity among the marginalized population is needed to increase maternal health service coverage.

## 1. Background

**Figure 1:**
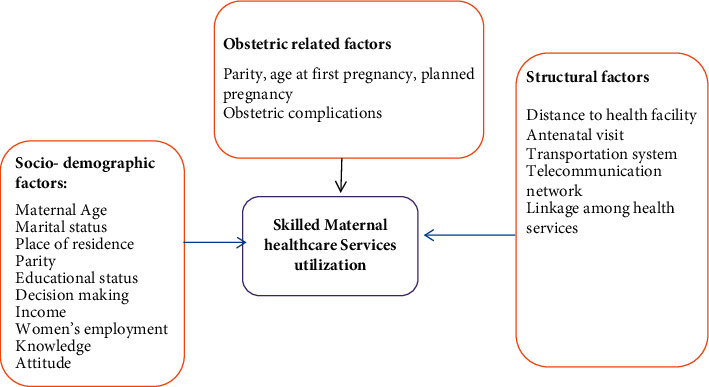
Conceptual framework adapted from different literature studies [5–10].

Access to skilled health services during pregnancy, childbirth, and postpartum is a crucial element that promotes the health and wellbeing of the mother and newborn. However, maternal mortality and morbidity remains a substantial concern. Globally, an estimated 303 000 mothers died due to maternal causes during pregnancy and childbirth and postpartum [1]. Developing regions account for 99% (302,000) of the global maternal deaths and sub-Saharan Africa accounts for two-thirds (201,000) [2]. Ending preventable maternal mortality by reducing the maternal deaths to less than 710 per 100,000 live births by 2030 requires rigorous improvements in skilled maternal healthcare [3]. However, only half of women in developing regions receive the amount of healthcare services they need. Currently, the Ethiopian government has made considerable progress in reducing maternal mortality. According to the Ethiopian Demographic Health Survey report, maternal mortality ratio has declined from 676 in 2011 to 412 in 2016 per 100,000 live births [4]. Despite improvements in maternal healthcare, there are still significant barriers to access and low utilization of maternal health services. In Ethiopia, an estimated 2.9 million women give birth every year and of these only 62%, 28%, and 17% of women received skilled antenatal care, skilled birth attendants, and postnatal care, respectively [4]. It is evident that maternal healthcare services are the most important interventions to prevent maternal morbidity and mortality but access to care alone is not enough to improve maternal health outcomes. Poor infrastructure, low quality care, and inequality substantially downplay efforts to escalate maternity services in low- and middle-income countries [5]. There are several factors influencing skilled maternal health services utilizations. Previous literatures show that these factors can be assorted as individual (sociodemographic and obstetric factors) and structural level (Figure 1). At the individual level, maternal level of education and awareness about skilled providers are perpetual predictors of antenatal care [5–7, 11–13]. Conversely, unplanned pregnancy and women giving birth more than once (multiparous) were less likely to utilize antenatal care [14]. At structural level, shortage of basic infrastructures, such as transportation facilities and telecommunications networks, significantly affected access to antenatal care services [8–10]. Previous studies in sub-Saharan Africa have revealed that education, women giving birth once (primiparous), previous experience of antenatal care visits, and awareness about skilled providers significantly predictors of skilled delivery [5–7, 11, 12, 15–21]. On the other hand, other studies demonstrate that multiparous had a positive effect on institutional delivery [22, 23]. Furthermore, evidence indicates that antenatal care attendance, wanted pregnancy, and birth complications were strong determinants of postnatal care services utilization [17, 24–26].

The government of Ethiopia plans to reduce maternal mortality, infant mortality, and morbidity by strengthening maternal healthcare system interventions essentially increasing birth attendants at birth, meeting unmet needs of family planning, improving quality of care at childbirth, and increasing financing of the health system, but still, maternal mortality is an unfinished issue which needs more investigations [27]. Nonetheless, there are many studies conducted on the utilization of maternal health services in Ethiopia [6, 7, 11, 15, 18, 20], but few studies have substantially addressed the women's level of knowledge and attitude regarding skilled maternal health services. Moreover, scant studies were done at community level in the West Shoa Zone. Therefore, this study assesses the women's knowledge, attitude, and practice of antenatal care, skilled birth attendants, and postnatal care and the associated factors in the West Shoa Zone, Ethiopia.

## 2. Methods

### 2.1. Study Area, Period, and Design

The study was conducted in West Shoa Zone, Oromia Region, Ethiopia. West Shoa is among 18 zones in Oromia region. The administrative center for West Shoa Zone is Ambo city which is located 114 km west of Addis Ababa, the capital city of Ethiopia. The West Shoa Zone has a total population of 2,058,676 of whom 1,028,501 were men and 1,255,010 were women of reproductive age [28]. The zone has 92 health centers, 578 health posts, 3 general hospitals, 4 district hospitals, and one referral hospital. The study was conducted from 1 February to 23 March 2018. Cross-sectional survey design was employed.

### 2.2. Source Population and Study Population

All women who gave birth in the last 12 months in West Shoa Zone were source of population and all randomly selected women who gave birth in the last 12 months in West Shoa Zone were study population.

### 2.3. Sample Size Determination and Sampling Procedure

The sample size was calculated using single population proportion formula [(*n* = (*Zά*/2)^2^*p*(1−*p*)/d^2^)] using a proportion of mother's seeking behavior, *P*=73.8% [29], with 5% of marginal error (d) and 95% confidence interval (CI), design effect of 2 to correct the design effect, and 10% nonresponse rate, yielding final sample size of 654. Simple random sampling technique was used to select the study participants.

**Figure 2:**
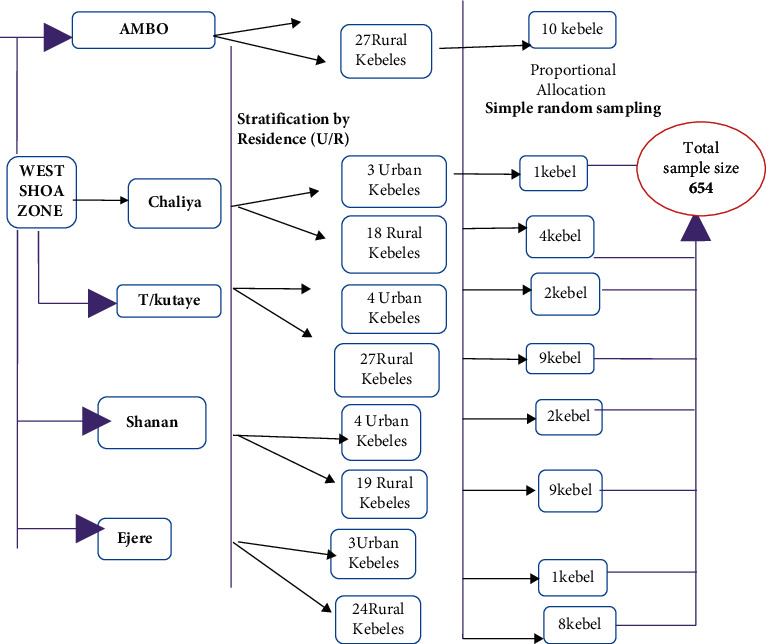
Schematic representation of women of reproductive age, West Shoa Zone, Ethiopia, 2018.

The regions are divided into zones, woredas, and kebeles which are the lowest level of administration. The woreda is administrative divisions with an average 100,000 population residing, and kebeles are the smallest unit in the local government of Ethiopia [27]. According to the West Shoa administrative office, the West Shoa Zone is composed of 19 Woredas with 528 rural kebeles and 58 urban kebeles. First five woredas such as Cheliya, Toke Kutaye, Nono, Dire Enchini, and Ejerie were purposely selected from a total of nineteen woredas from the zone. Then the five woredas were stratified by residence (urban and rural kebeles), and then the kebeles of the five woredas were allocated proportionally. The list of eligible women was obtained from registration books of respective kebeles' administration offices. The sample size was distributed to the urban and rural kebeles proportionate to the size of their population (Figure 2).

### 2.4. Inclusion and Exclusion Criteria

Women of reproductive age of 15–49 years who gave birth one year before the survey and residing in the study area for at least six months were included in this study and women with physical and mental illness were excluded from the study.

### 2.5. Study Variables

The dependent variables in this study were antenatal care, skilled birth attendants, and postnatal care and the independent variables were sociodemographic, obstetric-related factors, and structural factors.

### 2.6. Operational Definitions

Knowledge of skilled maternal health services is defined such that women who scored above the mean of knowledge assessment questions were categorized as having good knowledge, and if they scored below the mean, they were considered as having poor knowledge. Attitude was measured by using Likert scale (1 = strongly agree, 2 = agree, 3 = disagree, and 4 = strongly disagree). Positive attitude was scored by participants who respond above the mean of the attitude assessment questions and if below the mean they were categorized as having negative attitude. Practice (antenatal care, skilled delivery, and postnatal care utilization) was measured such that participants who respond above the mean of the practice assessment question were considered as having good practice and if below the mean they were considered as having poor practice.

### 2.7. Data Collection Tools, Procedure, and Data Quality Assurance

A structured questionnaire was used to collect the data. The questionnaire consists of sociodemographic characteristic (mother's age, marital status, place of residence, income, occupation, women's education, and husband education), obstetric history (parity, age at first pregnancy, pregnancy planned, and antenatal care visit), and service-related factors (distance to facility, transport, and telephone access), and questions addressing the women's knowledge, attitude, and practice of skilled assistance seeking maternal healthcare services were items in the questionnaire. The following measures were undertaken to assure the quality of data. The questionnaire was initially prepared in English, translated to the local language Afan Oromo and back to English by different individuals to check for consistency of meaning. The questionnaire was pretested on 33 women of reproductive age who were not participants in this study and lived outside the study area. Cronbach's alpha coefficient was used to ensure the reliability of the tools [30] and was found to be 0.89. Content validity was ensured by measuring content validity ratio and was 0.2. Then authors confirmed all items measured the content they were intended to measure. Six BSc nurse/midwife data collectors were recruited. Training was given to the data collectors for two days about the aim of the study, sampling procedures, and collecting the questionnaire data. The structured questionnaire was discussed in detail going through every question and clarification was provided. Informed consent was obtained to ensure the willingness and confidentiality of all of the study subjects. Then the collected data was reviewed and cross-checked for completeness and consistency by the principal investigator on daily basis at the spot during the data collection time.

### 2.8. Data Processing and Analysis

Data were entered and cleaned using EpiData version 3.1 software and then exported to SPSS version 20.0 statistical software packages for analysis. Bivariate and multivariate analysis between dependent and independent variables were performed separately using binary logistic regression. Descriptive statistics such as mean, median, and standard deviation were computed. Bivariate and multivariate logistic regression analysis were employed to examine the statistical association between independent and dependent variables. Variables that have a statistical association in the bivariate logistic regression at *P*-value <0.25 at 95% CI were entered into a multivariate logistic regression at *P*-value <0.25 at 95% CI [31]. Finally, adjusted odds ratio (AOR) with 95% CI and value <0.05 were considered statistically significant. Lastly, the results were presented using tables, figures, and texts.

## 3. Results

### 3.1. Sociodemographic Characteristics and Obstetric History of Study Participants

A total of 654 participants were enrolled in this study. The mean age of the study participants was 26.12 years. The study found that 405 (61.9%) of the participants were living in rural areas. The dominant ethnicity in the study area was Oromo (568—86.9%).

Concerning the marital status of the participants, 583 (89.1%) of women were married. Most of the women's educational status was grade 1 up to grade 8 which was 266 (40.7%). Among occupations, 181 (27.7%) were farmers and 197 (30.1%) women were housewives. The median monthly income of the women was <500 Ethiopian birr. In regard to the number of children, 477 (72.9%) of mothers have 2–4 children. Regarding overall women's age during their last recent birth, 323 (49.4%) were at the age of 15–19 years and 139 (25.5%) were at the age of 20–24 years. Regarding pregnancy, 541 (82.7%) of the participants had planned their last pregnancy. In the case of history of pregnancy and intrapartum complications, 376 (57.5%) had experienced complications in their last pregnancy and 223 (34.1%) of the women had encountered at least one complication during labor, out of whom 112 (50.2%) had excessive vaginal bleeding (Table 1).

### 3.2. Knowledge of Skilled Assistance Seeking Maternal Healthcare Services of Study Participants

The study found that, with respect to the knowledge score towards skilled maternal health services, 473.3 (72.4%) of the participants had good knowledge and 180.7 (27.6%) had poor knowledge towards skilled maternal health services. Regarding maternal health services information, 632 (96.0%) of the study participants had heard about skilled maternal health services and 265 (40.5%) health professionals were their main sources of information. Considering safety, 550 (84.1%) knew institutional delivery was safe, while 104 (15.9%) mentioned home delivery was safe. Regarding the knowledge of identifying skilled providers, 558 (85.3%) participants mentioned that health professionals are skilled providers, 84 (12.8%) mentioned that traditional birth attendants are skilled providers, and 12 (1.8%) participants mentioned that relatives are skilled providers. Regarding the apprehensions of the importance of postnatal care services, 477 (72.9%) of the study participants knew that postnatal care was important, and 177 (27.1%) knew that postnatal care was not an important service (Table 2).

### 3.3. Attitude of Skilled Assistance Seeking Maternal Healthcare Services of Study Participants

Regarding the attitude score on the need for skilled maternal care, 400 (61.2%) of the study participants had positive attitude towards skilled maternal health services, and 254 (38.84%) had negative attitude (Table 3).

### 3.4. Practice of Skilled Assistance Seeking Maternal Healthcare Services of Study Participants

**Figure 3:**
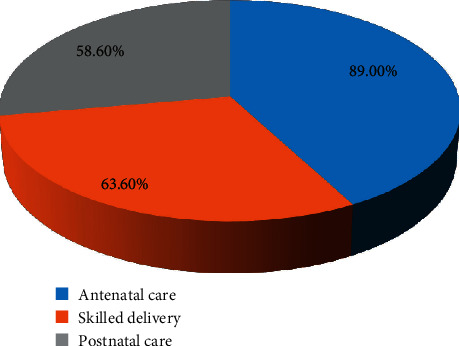
Practice of maternal health service utilization among women of reproductive age at West Shoa Zone, Ethiopia, 2018.

Based on practice score, 460.3 (70.4%) of the participants had good practice and 193.7 (29.6%) had poor practice towards skilled maternal health services. Regarding the utilization of antenatal care, 582 (89%) of the women had an antenatal checkup, of whom 249 (42.8%) participants had four and above antenatal checkups. Regarding the place of childbirth delivery, 416 (63.6%) of participants attended their recent childbirth in health facilities by skilled birth attendant, and 238 (36.4%) gave birth at home (Figure 3). Out of those women who gave birth at home, 95 (39.7%) were assisted by traditional birth attendants. Regarding the reason for home delivery, 101 (42.2%) experienced urgent labor, 92 (38.5%) had usual childbirth experiences, 27 (11.3%) had distant health facilities, 14 (5.9%) depended on presence of traditional birth attendants, 5 (2.1%) lacked transportation, and 376 (57.5%) encountered birth complications in their recent childbirth. Among those who gave birth at health institutions, 383 (58.6%) women had received postnatal care at health facilities (Table 4).

### 3.5. Factors Associated with Skilled Assistance Seeking Antenatal Care Services of Study Participants

On multivariate analysis, planned pregnancy and access to transport were found to be significantly associated with antenatal care utilizations. Women who had a planned pregnancy were eight times more likely to seek antenatal care than unplanned pregnancy (AOR = 8.2, 95% CI = 3.39-19.78-0.87), women who had access to transportation were three times more likely to seek skilled antenatal care than those who had no transportation access (AOR = 3.1, 95% CI = 1.46–6.61) (Table 5).

### 3.6. Factors Associated with Skilled Assistance Seeking Delivery Services of Study Participants

In multivariate analysis, women's education, wanted pregnancies, parity, antenatal care visit at least once, experiencing birth complications, and knowledge about skilled delivery were found to be statistically significant with skilled assistance seeking delivery services. The study found that education increases the probability of women utilizing skilled maternal healthcare services. Women with educational level of secondary and above (AOR = 3.0, 95% CI = 1.18–7.84) were three times more likely to have childbirth at the health facility as compared to those women who had no formal education, women whose previous pregnancies were unwanted had 70% lower odds of attending childbirth at the health facility as compared to those women with wanted pregnancies (AOR = 0.3, 95% CI = 0.21–0.75), primiparous women had 89% lower odds of attending childbirth at health facility than the multiparous women (AOR = 0.11, 95% CI = 0.05–0.24), women having at least one antenatal care in their recent pregnancies were about three times more likely to attend childbirth by a skilled provider compared with those who had no antenatal visit (AOR = 3.1, 95% CI = 1.13–8.41), women who had experienced birth complications were twice more likely to seek skilled provider than those who had not had complications (AOR = 2.3, 95% CI = 1.39–3.75), and women who had awareness about skilled obstetric care were three times more likely to have birth attendance by a skilled provider with their counterparts (AOR = 3.1, 95% CI = 1.13–8.41) (Table 6).

### 3.7. Factors Associated with Skilled Assistance Seeking Postnatal Services of Study Participants

In multivariate analysis, number of antenatal care visits, pregnancy complications, unwanted pregnancies, and having awareness about skilled obstetric care were significantly associated with postnatal care by a skilled provider. Women having at least one ANC in their recent pregnancy were twice more likely to attend postnatal care as compared with those who had no antenatal visit (AOR = 2.1, 95% CI = 1.1–4.2.), women who had experienced birth complications were twice more likely to seek postnatal care than those who had not had complications (AOR = 2.2, 95% CI = 1.35–3.66), women with unwanted pregnancies had 70% lower odds of attending postnatal care services as compared to women of wanted pregnancies (AOR = 0.3, 95% CI = 0.22–0.68), and women who had awareness about skilled obstetric care were four times more likely to attend postnatal care with their counterparts (AOR = 3.7, 95% CI = 1.68–12.79) (Table 7).

## 4. Discussion

This study assessed the women's knowledge, attitude, and practice of skilled assistance seeking maternal healthcare services. In this study, the proportion of antenatal care, skilled delivery, and postnatal care services utilization was low as compared to other studies [21, 22, 25]. Conversely, the institutional delivery in this study was found high as compared to other studies in Ethiopia and Kenya [7, 12, 13, 16]. The reasons for this variation could be explained by the different sample sizes, time gaps, and different socioeconomic conditions of the settings.

Among the predisposing factors, access to transport and planned pregnancy were found to be associated with the use of antenatal care. Transport access is often reported readily available in the study settings which delays women's timely healthcare. The findings suggest that women who had access to transport were more likely to seek antenatal care service than those women who had no transport access. This implies that basic infrastructure inevitably has an effect on antenatal care utilization. This finding is consistent with the study done in Ghana, Kenya, and Malawi emphasising that the availability of vehicles such as public transport and taxis significantly determined the pregnant women's decision to seek antenatal care [10]. Furthermore, other studies in sub-Saharan Africa also ascertained that access to transport services plays a critical role in women's antenatal care attendance [8, 9]. Moreover, this study found that planned pregnancies were significantly associated with antenatal care utilization. However, this finding is congruent with a study in the Democratic Republic of Congo [14].

Regarding the predisposing factors to skilled delivery, women's education was significantly associated with skilled delivery utilization. Women with secondary school and above were more likely to deliver at a health facility as compared to women with no education. The findings of this study are similar with other studies in Africa [5–7, 11, 12] which highlighted that utilization of maternal health services increases consistently as the educational level increases. The higher utilization of skilled childbirth services among well-educated women may be attributed to their level of understanding, which may make women who are more concerned about their health and their illness need to seek appropriate healthcare services. Furthermore, women with unplanned pregnancies were 70% less likely to have childbirth at the health facility as compared to those mothers with wanted pregnancies. This finding is also supported by a study in the Democratic Republic of Congo [14]. This might be because the occurrence of unintended pregnancy is likely to reduce maternity care-seeking behavior of women, which is associated with discouragement and feeling less pregnancy experience.

Our finding showed that parity is significantly associated with a skilled birth attendant. Primiparous women were more likely to deliver in a health facility than the multipara. This finding is consistent with other studies done in Kenya and Ethiopia [16, 20]. This might be because the low parity women give more attention to childbirth experiences and might have fear of complications than high parity women. This finding is in contrast with the previous studies in Ethiopia and Nigeria [22, 23]. Women who had experienced birth complications were found to have a significant association with seeking skilled delivery. This finding is also supported by other studies in the Oromia region of Ethiopia [18]. Access to information on the importance of skilled maternal healthcare is also associated with the utilization of skilled birth attendants. This finding is similar to the study done in Ethiopia [13]. The number of antenatal care visits tended to increase the utilization of skilled delivery. This study has found that women who had to attend at least one antenatal care for their previous pregnancies were more likely to seek skilled delivery compared with those who did not have antenatal care visits. This finding is similar to that of studies in Ethiopia, Tanzania, South Sudan, and Nepal, respectively [15, 17, 19, 21]. This might be because antenatal care is a significant intervention in contributing women into contact with the health system, facilitating women's access to skilled childbirth and including postnatal care. This implies that undergoing constant antenatal care visits have predominant importance to increase the utilization of facility delivery services.

This study illustrates that having at least one antenatal care visit in women's recent pregnancies was a significant predictor of postnatal care services. This finding is supported by other studies in Nigeria [26]. Likewise, wanted pregnancies were significantly associated with the postnatal care utilization. Women of unwanted pregnancies were less likely to attend postnatal care services compared to women of wanted pregnancies. This is consistent with the study done in Tanzania [17]. This implies that unwanted pregnancy influences maternal healthcare services. The study found that experiencing obstetric complications was a significant predictor to seek postnatal care. For instance, women tend to visit the health facility for postnatal care only when they encountered complications. This implies that postnatal care services do not give much attention in the study area. This finding also agrees with a study conducted in Nepal and Tanzania [24, 25].

### 4.1. Limitation of the Study

The study has encountered certain limitations. The study used a cross-sectional study design that has considerable methodological limitations in drawing cause and effect relationships between the variables. The information obtained from the participants could be affected by social desirability due to recall bias; thus the study attempts to minimize this by including women who gave birth in the last year.

## 5. Conclusions

This study found that the knowledge, attitude, and practice of the study participants towards skilled maternal health services are low, which is less than three-quarters of the total sample size. Therefore, the findings of this study indicate that interventions are required to improve women's knowledge, attitude, and practice of skilled maternal health services in the study area. Moreover, unplanned pregnancy and lack of transportation were significantly associated with the nonutilization of maternal health services. Therefore, the study suggests that integrated family planning and maternal healthcare services should be reconsidered to assist women with unplanned pregnancies to utilize maternal healthcare services and improvement to infrastructures are needed to increase access to maternal health services. Likewise, women's education is significantly associated with skilled delivery services utilization. Accordingly, this study recommends that improving equity among the marginalized population is needed to increase maternal health services coverage.

## Figures and Tables

**Table 1:** Sociodemographic characteristics of study participants.

Variable	Category	Frequency	Percentage
Residence	Rural	405	61.9
	Urban	249	38.1

Age	15–19	21	3.2
	20–24	137	20.9
	25–29	262	40.1
	30–34	152	23.2
	35–39	69	10.6
	40–44	11	1.7
	45–49	2	0.3

Marital status	Single	15	2.3
	Married	583	89.1
	Divorced	37	5.7
	Divorced	19	2.9

Educational status of the mother	Unable to read and write	171	26.1
	Grade (1–8)	266	40.7
	Grade (9–12)	139	21.3
	College and above	78	11.9

Occupation	Farmer	181	27.7
	Housewives	197	30.1
	Daily worker	41	6.3
	Merchant	111	17.0
	Office worker	108	16.5

Income	1–500 birr	419	64.1
	501–1000 birr	141	21.6
	>1000 birr	87	13.3
	None	7	1.1

Number of children	1	97	14.8
	2–4	477	72.9
	≥5	80	12.2

Last pregnancy planned	Yes	541	82.7
	No	113	17.3

Pregnancy complication	Yes	376	57.5
	No	278	42.5

**Table 2:** Knowledge towards skilled maternal healthcare among study participants.

Variable	Category	Frequency	Percentage
Ever heard about skilled maternal health services?	Yes	628	96.0
	No	26	4.0

Source of information about skilled maternity care?	Friends	92	14.1
	HEW	160	24.5
	Media-radio/TV	31	4.7
	Families	80	12.2
	Health professionals	265	40.5

Know every pregnant mother should receive antenatal care?	Yes	632	96.6
	No	22	3.4

Which is safe for child delivery?	Health facility	550	84.1
	Home delivery	104	15.9

Which provider is skilled for delivery?	Health professional	558	85.3
	TBA	84	12.8
	Relatives	12	1.8

Know postnatal care is important?	Yes	477	72.9
	No	177	27.1

**Table 3:** Attitude towards skilled maternal healthcare among study participants.

Variable	Strongly agree	Agree	Disagree	Strongly disagree
Do you agree the importance of skilled health providers for maternity care?	442 (67.6)	212 (32.4)	—	—
How do you agree that the need of having a plan on possible pregnancy complication?	214 (32.7)	432 (66.1)	8 (1.2)	—
Do you agree delays in seeking care for obstetric complication contribute to maternal death?	83 (12.7)	325 (49.7)	234 (35.8)	12 (1.8)
How do you agree to the importance of planning delivery place?	149 (32.8)	498 (76.1)	7 (1.1)	—

**Table 4:** Practice towards skilled maternal healthcare among study participants.

Variable	Category	Frequency	Percentage
Attend antenatal care for last pregnancy?	Yes	582	89.0
	No	72	11.0

Number of antenatal care visits	1	20	3.4
	2–3	313	53.8
	4 and above	249	42.8

Place of delivery	Home	238	36.4
	Health facility	416	63.6

Delivery assisted by	Doctor	35	5.4
	Nurse	83	12.7
	Midwives	280	42.8
	Health officer	8	1.2
	I don't remember	9	1.4

Home assisted by	Traditional birth attendants	95	39.7
	Neighbours	88	36.8
	Relatives	51	7.8
	Health extension workers	5	2.1
	Usual experience	92	38.5
	Labor is urgent	101	42.2

Reasons for home delivery	Presence of traditional birth attendants	14	5.9
	Health facilities are far away	27	11.3
	Lack of transportation	5	2.1

Birth outcome	Live birth	630	96.3
	Still birth	24	3.7

Did you attend postnatal care from health facility for last pregnancy?	Yes No	383 271	58.6 41.4

Experienced obstetric problem the last pregnancy?	Yes	376	57.5
	No	278	42.5

**Table 5:** Binary logistic regression model to examine the association of antenatal care services among study participants.

Variable	Category	Seek ANC		Crude OR (95% CI)	*P* value	Adjusted OR (95% CI)	*P* value
		Yes	No				
Age at last delivery	15–19	17	4	0.56 (0.01–44.49)	0.56	—	—
	20–24	131	6	0.04 (0.03–0.82)	0.04	1	—

Marital status	Single	9	6	1.2 (0.12–12.5)	0.8	—	—
	Married	536	47	0.06 (0.02–0.66)	0.000	1	—

Mother's education	No formal education	141	30	1.0 (0.59–17.15)	0.99	—	—
	Formal education	107	33	10.9 (1.46–81.1)	0.02	1	—
Husband's education	No formal education	107	17	2.0 (0.36–11.18)	0.42	—	—
	Formal education	203	30	0.09 (0.04–0.25)	0.000	1	—

Income	<500 birr	86	1	3.7 (0.11–121.75)	0.46	—	—
	>1000 birr	366	53	0.03 (0.002–0.38)	0.007	1	—

Planned pregnancy	Yes	513	28	0.1 (0.05–0.15)	0.08	8.2 (3.39-19.78)^*∗*^	0.001
	No	69	44	—	—	—	—

Number of children	1	89	8	0.7 (0.14–3.96)	0.74	—	—
	2–4	440	37	0.1 (0.09–0.29)	0.000	1	—

Transport access	Yes	298	25	0.5 (0.3–0.85)	0.01	3.1 (1.46-6.61)^*∗*^	0.003
	No	284	47	—	—	—	—

Knowledge about skilled maternity care	Yes	565	63	0.2 (0.09–0.49)	0.000	1.9 (0.04-0.87)^*∗*^	0.01
	No	17	9	—	—	—	—

Attitude about skilled maternity care	Good	408	34	0.38 (0.23–0.63)	0.000	1	—
	Poor	174	38	—	—	—	—

Significant for *P* value <0.05; ^*∗*^statistically significant for *P* value ≤0.01.

**Table 6:** Binary logistic regression model to examine the association of delivery services among study participants.

Variable	Category	Seek skilled delivery		Crude OR (95% CI)	*P* value	Adjusted OR (95% CI)	*P* value
		Yes	No				
Residence	Rural	248	157	1.3 (0.94–1.83)	0.1	—	—
	Urban	168	81	—	—	—	—

Marital status	Single	9	6	0.1 (0.04–0.81)	0.025	—	—
	Married	384	199	0.1 (0.004–0.42)	0.001	—	—

Mother's education	Unable to read and write	88	83	6.4 (3.0–13.28)	0.000	—	—
	Grades 9–12, college	98	41	2.8 (1.3–6.06)	0.007	3.0 (1.18–7.84)^*∗*^	0.02

Husband's education	Unable to read and write	76	48	0.5 (0.26–1.1)	0.09	—	—
	Grades 9–12	93	33	0.3 (0.14–0.64)	0.002	—	—
	College	110	22	0.1 (0.07–0.37)	0.000	—	—

Planned pregnancy	Yes	382	159	0.17 (0.11–0.28)	0.000	0.3 (0.21–0.75)^*∗*^	0.004
	No	34	79	—	—	—	—

Number of children	1	13	7	0.04 (0.02–0.09)	0.00	—	—
	2–4	221	92	0.09 (0.05–0.17)	0.00	0.11 (0.05–0.24)^*∗*^	0.001

Antenatal care attend	Yes	389	193	0.29 (0.17–0.49)	0.000	1	
	No	27	45	—	—	—	—

Number of antenatal care visits	At least once	13	7	2.7 (1.59–4.72)	0.000	3.1 (1.13–8.41)^*∗*^	0.03
	2 and above	221	92	0.6 (0.48–0.97)	0.04	—	—

Experienced complication	Yes	205	171	2.6 (1.86–3.69)	0.000	4.7 (2.7–8.43)^*∗*^	0.001
	No	211	67	—	—	—	—

Transport access	Yes	214	109	0.7 (0.58–1.09)	0.16	1	
	No	202	129	—	—	—	—

Knowledge about skilled delivery	Yes	407	221	0.28 (0.13–0.65)	0.003	3.1 (1.13–8.41)	0.03
	No	9	17	—	—	—	—

Attitude about skilled delivery	Positive	305	111	0.4 (0.35–0.69)	0.000	1	—
	Negative	137	101	—	—	—	—

Significant for *P* value <0.05; ^*∗*^statistically significant for *P* value ≤0.01.

**Table 7:** Binary logistic regression model to examine the association of postnatal care services among study participants.

Variable	Category	Seek PNC		COR (95% CI)	*P* value	AOR (95% CI)	*P* value
		Yes	No				
Number of children	2–4	385	92	0.34 (0.1–0.83)	0.01	1	—
Number of antenatal care visits	4 & above	184	65	0.44 (0.23–0.84)	0.01	2.1 (1.1–4.2)^*∗*^	0.025
Transport access	Yes	249	74	3.3 (1.39–8.0)	0.007	1	—
Experienced pregnancy complication	Yes	256	120	2.2 (1.0–5.14)	0.04	2.2 (1.35–3.66)^*∗*^	0.002
Source of information about skilled providers	Health professional	231	34	2.3 (1.03–5.07)	0.04	3.7 (1.68–12.79)^*∗*^	0.003
Planned pregnancy	Yes	454	87	0.3 (0.14–0.66)	0.003	0.3 (0.22–0.68)^*∗*^	0.001

Significant for *P* value <0.05; ^*∗*^ statistically significant for *P* value ≤0.01.

## Data Availability

The datasets used during the current study are available from the corresponding author upon reasonable request.
